# Optimizing the Use of Electronic Health Records to Identify High-Risk Psychosocial Determinants of Health

**DOI:** 10.2196/medinform.8240

**Published:** 2017-08-14

**Authors:** Nicolas Michel Oreskovic, Jennifer Maniates, Jeffrey Weilburg, Garry Choy

**Affiliations:** ^1^ Internal Medicine and Pediatrics Massachusetts General Hospital Boston, MA United States; ^2^ Harvard Medical School Boston, MA United States; ^3^ Integrated Care Management Program Massachusetts General Hospital Boston, MA United States; ^4^ Department of Psychiatry Massachusetts General Hospital Boston, MA United States; ^5^ Department of Radiology Massachusetts General Hospital Boston, MA United States

**Keywords:** word recognition, Medicaid, psychosocial determinants of health, social determinants of health, care coordination

## Abstract

**Background:**

Care coordination programs have traditionally focused on medically complex patients, identifying patients that qualify by analyzing formatted clinical data and claims data. However, not all clinically relevant data reside in claims and formatted data. Recently, there has been increasing interest in including patients with complex psychosocial determinants of health in care coordination programs. Psychosocial risk factors, including social determinants of health, mental health disorders, and substance abuse disorders, are less amenable to rapid and systematic data analyses, as these data are often not collected or stored as formatted data, and due to US Health Insurance Portability and Accountability Act (HIPAA) regulations are often not available as claims data.

**Objective:**

The objective of our study was to develop a systematic approach using word recognition software to identifying psychosocial risk factors within any part of a patient’s electronic health record (EHR).

**Methods:**

We used QPID (Queriable Patient Inference Dossier), an ontology-driven word recognition software, to scan adult patients’ EHRs to identify terms predicting a high-risk patient suitable to be followed in a care coordination program in Massachusetts, USA. Search terms identified high-risk conditions in patients known to be enrolled in a care coordination program, and were then tested against control patients. We calculated precision, recall, and balanced F-measure for the search terms.

**Results:**

We identified 22 EHR-available search terms to define psychosocial high-risk status; the presence of 9 or more of these terms predicted that a patient would meet inclusion criteria for a care coordination program. Precision was .80, recall .98, and balanced F-measure .88 for the identified terms. For adult patients insured by Medicaid and enrolled in the program, a mean of 14 terms (interquartile range [IQR] 11-18) were present as identified by the search tool, ranging from 2 to 22 terms. For patients enrolled in the program but not insured by Medicaid, a mean of 6 terms (IQR 3-8) were present as identified by the search tool, ranging from 1 to 21.

**Conclusions:**

Selected informatics tools such as word recognition software can be leveraged to improve health care delivery, such as an EHR-based protocol that identifies psychosocially complex patients eligible for enrollment in a care coordination program.

## Introduction

An increasing number of states in the United States are transitioning from fee-for-service care to establishing accountable care organizations (ACOs) for patients enrolled in Medicaid, a health care program for people with limited resources, in efforts to improve patient outcomes and control health care costs. Since 2012, 14 states have developed Medicaid ACOs, with Massachusetts launching a pilot version in December 2016 [1]. With the prospect of both Medicaid and Medicare patients enrolled in ACOs in large health care networks across many states, such as Massachusetts, accountability for risk and quality will increasingly be assumed by health care networks and participating providers. For many patients enrolled in Medicaid ACOs, managing risk and improving outcome markers will require understanding factors other than traditional medical complexity [2]. Patients enrolled in Medicaid can often have a variety of upstream social factors that can influence their health, such as housing and employment instability and food insecurity, collectively known as social determinants of health, as well as mental health conditions and substance abuse. These psychosocial factors can shape one’s ability to obtain health needs and adhere to health recommendations, and can have a substantial impact on health outcomes [3]. The ability of health care networks participating in Medicaid ACOs to identify those patients with psychosocial drivers with the highest utilization will become increasingly important as networks seek to contain escalating health care costs and appropriately manage pooled risk [4].

While various approaches to identifying medical complexity from an electronic health record (EHR) have been developed and are being employed by health care networks across the United States, there is less certainty about how to identify and grade psychosocial complexity from an EHR [5,6]. As with patients with a high degree of medical complexity, patients with high psychosocial complexity may likewise use and consume substantial health care resources and be challenging to manage clinically [7,8]. Accordingly, there may be value in developing an EHR-based data mining tool for identifying patients with increased psychosocial complexity. Once identified, such patients could be enrolled in a care coordination program that manages complex patients and focuses on decreasing health care utilization and containing health care costs. Care coordination programs have traditionally cared for medically complex patients and have developed various approaches to identifying patients who qualify as high risk [9]. Unlike medical complexity, psychosocial complexity may be more difficult to identify. Medically complex patients are typically identified using International Classification of Diseases codes from claims data or EHR-based algorithms that use structured fields in the medical chart (diagnosis codes, problem lists, medications, or laboratory studies). Privacy laws around mental health and substance abuse, along with the lack of formatted fields for many of the risk factors underlying psychosocial risk, make identifying patients with high psychosocial complexity more challenging. The data necessary to populate the risk categories are often unavailable or suboptimal for population-level screening. Furthermore, when compared with more automated and search technology-enabled approaches, individual chart review is impractical given its time-consuming and often subjective nature of identifying patients with high complexity.

Given the known limitations and challenges of using available data to identify patients with increased psychosocial risk, we sought to develop an EHR-based tool that could identify patients with increased psychosocial risk. We used the analytics platform QPID (Queriable Patient Inference Dossier; developed at Massachusetts General Hospital and QPID Health Inc, Boston, MA, USA) to search the EHR for key terms predictive of psychosocial risk. QPID is a health intelligence platform incorporating an EHR search engine with a scalable library of US Health Insurance Portability and Accountability Act (HIPAA) -compliant search queries, and a programmable ontology-driven system for application and query development [10]. The engine searches all the data residing within a patient’s EHR, including inpatient and outpatient notes, radiology reports, and laboratory data, and can be used to extract detailed information from a single patient’s EHR or can be run against an entire patient census.

QPID consumes both structured and unstructured data from the EHR. The unstructured data are in free-text form from the medical record in native format. Both forms of data are extracted, transformed, and loaded into the QPID system, which then performs natural language processing, term indexing, and data aggregation to find and combine medically relevant entities for patients and populations. The natural language processing involves negation detection and date detection, among other techniques. A querying language is overlaid on this processed data to access and visualize data as needed. Medical concepts are clustered through structured ontologies and machine learning techniques, and both open-source ontologies and proprietary clinical knowledge mappings are used. The terms can be mapped to Medical Subject Headings (MeSH); however, custom mapping of medical concepts is often necessary to supplement existing ontologies, especially in the space of psychosocial factors, given that general ontologies often only include biomedically relevant concepts and may lack the nuance to capture social aspects of a patient’s well-being.

We hypothesized that, using programmable word recognition software, we could identify patients with high psychosocial complexity at risk for increased health care utilization by using only data available in a patient’s EHR.

## Methods

### Study Population

The study included patients receiving care at Massachusetts General Hospital, a major academic medical center located in Boston, MA, USA. We analyzed EHRs of 132 patients covered by Medicare using QPID to determine the validity of the 22 search terms that we identified. We tested the algorithm on 120 patients enrolled in a care coordination program with documented risk profiles and known psychosocial complexity. Of these 120 index patients, 60 were enrolled in a Medicaid insurance program and 60 were not enrolled in Medicaid. The Medicare patients served as real-world controls against the Medicaid patients, with known higher rates of psychosocial comorbidity. The Impact Pro score—a medical risk-predictive modeling score based on medical and pharmacy claims data and medical diagnoses information—was available for all patients enrolled in the care coordination program. An additional 12 healthy patients not enrolled in a care coordination program or Medicaid, of whom 6 were adults and 6 were children, served as true-negative controls.

### QPID

We used QPID to search patients’ EHRs for terms associated with underlying clinical conditions and social risk factors. A list of 54 terms belonging to 4 psychosocial domains (mental health, substance use, social determinants, and legal history) was generated, from which we ultimately identified 22 terms as being sufficiently sensitive and specific to the clinical or social marker being queried (see below). As part of the search term algorithm development process, we removed certain terms that were sensitive but not specific, as summarized below. A blinded manual chart review without knowledge of the search term results was conducted for every study patient by 1 of the study investigators (NO) with expertise in care coordination, with a clinical determination based on clinical judgment for each patient on whether they required care coordination to help manage their psychosocial complexities. The chart review served as the reference standard for assessing psychosocial complexity and ensured that the 22 search terms correctly identified documented psychosocial risk and distinguished psychosocial from medical risk. Using the chart review and QPID result, we created a contingency table and assigned each patient to 1 of 4 categories: true positive, true negative, false positive, or false negative.

### Sensitive But Not Specific Search Terms

We designed several search queries (terms) to be sensitive markers (correctly identified patients who were at risk) but nonspecific (also identified patients without risk in whose chart the search term was present but not assigned to the index patient). A relatively more sensitive than specific search term was better suited for screening health records. Several scenarios produced false positives, including lexical variations such as polysemy (a term or abbreviation with multiple meanings), negation, preformatted text, and misallocation. In one example of polysemy, the search term “AA” was a useful and effective marker for identifying alcohol abuse by correctly identifying patients where AA was used as an abbreviation for Alcoholics Anonymous in the EHR, but infrequently also incorrectly identified charts where AA was used as shorthand to signify unrelated categories, including clinical information (amino acid) and demographic information (African American). Negation, a common finding and false-positive source, existed where the search term was listed in a patient note as not being present. Preformatted text was another scenario that produced sensitive but nonspecific terms, where, for example, the term “depression” incorrectly identified all screening questionnaires and preformatted notes in the EHR that included the word depression, even when a patient reported not being depressed. Misallocation was another scenario resulting in false positives, where data in the EHR describing the reported condition of a friend or relative were incorrectly assigned to the index patient, as with the term “arrested;” an example of this was the mention in the EHR of a patient’s son being arrested.

### Statistical Approach

We used descriptive statistics to calculate the number of times each term was present within a patient’s EHR, and report the mean, interquartile range (IQR), and range for each search term by patient group. We created the list of search terms in the final algorithm by including only terms where the IQRs for index and control patients did not overlap. We compared results between Medicaid-enrolled patients and non-Medicaid-enrolled patients (controls), the latter of which included both non-Medicaid index patients enrolled in the care coordination program and true-negative patients—that is, non-Medicaid patients not enrolled in the care coordination program. Using contingency table results, we calculated the accuracy, precision, recall, and balanced F-measure for the 22-term algorithm’s ability to correctly detect and assign psychosocial complexity.

## Results

Table 1 describes the study population, providing a summary of demographics and clinical information. Mean Impact Pro scores were not statistically different between Medicaid and non-Medicaid patients enrolled in the care coordination program (3.2 vs 6.2, *P*=.9).

We identified 22 search terms that correctly predicted increased psychosocial risk with a high degree of specificity: anxiety, depressed, sad, angry, neurovegetative, schizoaffective, substance, abuse, addict, aa, sober, cocaine, heroin, crack, mushrooms, prison, jail, homeless, shelter, stamps, stolen, and tox.

**Figure 1:**
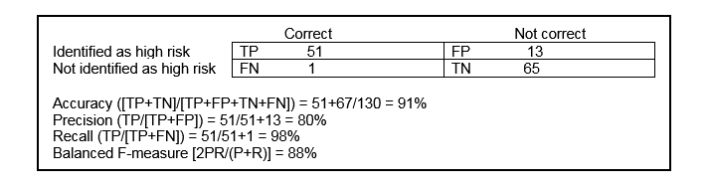
Performance evaluation using a contingency table of the 22-term QPID (Queriable Patient Inference Dossier) algorithm for identifying psychosocial complexity. FN: false negative; FP: false positive; TN: true negative; TP: true positive.

Among the 60 patients enrolled in Medicaid, the mean number of terms per patient was 14.1 (IQR 11-18, range 2-22). Among the 72 control patients not enrolled in Medicaid, the mean number of terms per patient was 6.0 (IQR 3-8, range 1-21). Among the true-negative patients, the mean number of terms per patient among pediatric patients was 2.7 (range 2-3), and among adult patients it was 2.0 (range 1-3). As Figure 1 shows, in blind testing, the 22-search term-based analysis achieved an overall 91% accuracy, 80% precision, 98% recall, and balanced F-measure of 88%.

**Figure 2:**
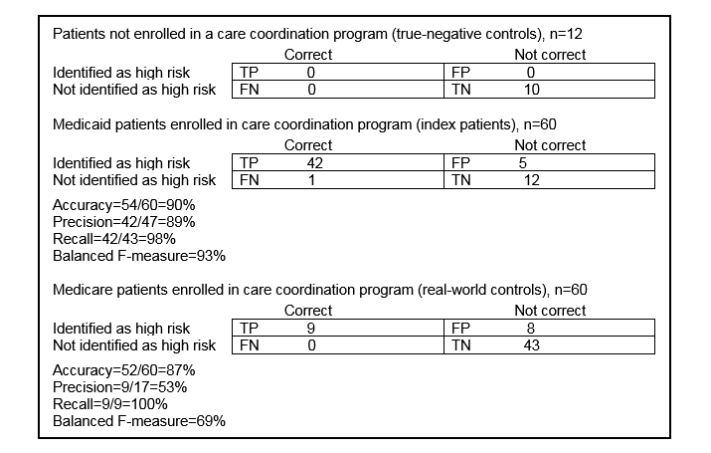
Performance evaluation using contingency tables of the 22-term QPID (Queriable Patient Inference Dossier) algorithm by insurance payer and care coordination status. FN: false negative; FP: false positive; TN: true negative; TP: true positive.

The 22-search term-based analysis performed well among both Medicare-enrolled and Medicaid-enrolled patients, as well as patients not enrolled in a care coordination program (Figure 2).

**Table 1:** Study population characteristics.

Characteristics		Index patients (care coordination program enrollees)		True negatives (non-Medicaid, noncare coordination program)	
		Medicaid (n=60)	Non-Medicaid (n=60)	Adults (n=6)	Children (n=6)
Age in years, mean		41	66	64	6
Male, n (%)		32 (53)	27 (45)	3 (50)	4 (67)
**Race/ethnicity, n %**					
	White	54 (90)	50 (83)	6 (100)	6 (100)
	Black	2 (3)	1 (2)	0	0
	Hispanic/Latino	3 (5)	4 (7)	0	0
	Other	1 (2)	5 (8)	0	0
Impact Pro^a^ score, median		3.2	6.2	N/A^b^	N/A
QPID^c^ terms, mean		14.1	6.0	2.0	2.7

^a^A medical risk-predictive modeling score that uses medical and pharmacy claims data, laboratory results, and medical diagnoses information to predict patients at risk for future severe health problems.

^b^N/A: not available.

^c^QPID: Queriable Patient Inference Dossier.

## Discussion

In this paper, we describe the use of a word recognition software program to develop a search term algorithm that accurately identifies Medicaid-enrolled patients with elevated psychosocial risk as distinct from medical risk. While methods exist for assessing and quantifying medical risk using existing medical taxonomy and medical insurance claims data, respectively, psychosocial risk, in contrast, is less well defined in medical claims data and not as robustly classified by medical nomenclature, making it harder to identify using existing datasets. With the expansion of Medicaid ACOs across the United States, and the known prevalence of psychosocial complexity among patients enrolled in Medicaid insurance programs, there will be increased pressure to identify increased psychosocial risk among Medicaid populations for population health management, as well as increasing demand for clinical decision support systems with the capacity to identify patient-attributable psychosocial risk concepts on an individual patient level [11]. Our novel approach offers the ability to use a patient’s EHR as a way to identify important psychosocial risk factors potentially driving or contributing to health care utilization and costs, and medical outcomes, among patients enrolled in Medicaid. Moreover, by running our model on patients followed in a care coordination program that manages patients with known medical and psychosocial complexity, we were able to use the algorithm to disentangle medical and psychosocial risk and identify those patients with active psychosocial complexity. In so doing, our findings also underscore the importance of understanding and accounting for psychosocial risk, and provide a mechanism through which providers and health care networks can assess and manage their risk pool by quantifying and triaging psychosocial risk.

Setting the positive criteria as having 9 or more terms present in the EHR as identified by our search tool allowed us to identify patients with a moderate to high burden of active psychosocial complexity, while excluding patients with an existing but low psychosocial complexity or patients with several false-positive markers. Creating an algorithm that assigns the outcome status based on a count of EHR-identified categories rather than on raw term counts avoids creating an algorithm that includes patients who may have a single domain of psychosocial complexity that is frequently documented (eg, a patient whose only health problem is severe anxiety requiring frequent health care visits) or a patient without any psychosocial complexity who has multiple false-positive data returns (eg, an elderly woman who has been administered multiple depression screens over the years; an adult patient with a remote history of child abuse frequently documented in the EHR). Another decision when building the algorithm was to not use date search parameters, given known limitations with how data are entered into and notes are formatted in the EHR (eg, old text sections frequently being carried over into new notes; pretyped templates containing false-positive terms).

Our study has several limitations worth noting. First, our study was a retrospective chart review, and did not prospectively predict outcomes or utilization. Second, we did not compare our findings with utilization data. Large categories of health care utilization data, including mental health data, are not available due to HIPAA requirements, making a valid cost analysis of psychosocial risk difficult to perform. Third, our reference standard for psychosocial complexity was inclusion in a care coordination program with documented psychosocial complexity requiring social work and mental health services. While possibly subjective and difficult to systematize, the advantage of using patients with known psychosocial complexity who receive services is that this approach uses real-world examples and results in an algorithm that can identify patients who can benefit from such services. Fourth, we used search terms as proxies for identifying clinical concepts, an approach that leverages the power of natural language processing software to search unformatted text for data retrieval; nevertheless, terms and concepts are not necessarily the same, and a clinical concept may be present even when search terms are not. Fifth, for the methods we describe in this paper to be scalable, the technology will require additional functional enhancements. We ran each patient’s data through QPID individually and manually counted the number of identified search items; in order for the approach we describe to be useful for large health care networks, one would need the ability to batch run a list of patients, and the software should automatically return term tallies for each patient.

Despite these limitations, this study provides an important step forward for population health management by outlining a new method for identifying the important role that social determinants and mental health play in health outcomes, and offers a promising new approach to stratifying this risk burden on a population level.

## Abbreviations

ACO: accountable care organization
EHR: electronic health record
HIPAA: Health Insurance Portability and Accountability Act
IQR: interquartile range
MeSH: Medical Subject Headings
QPID: Queriable Patient Inference Dossier
